# Aberrant CDK9 expression within chordoma tissues and the therapeutic potential of a selective CDK9 inhibitor LDC000067

**DOI:** 10.7150/jca.35426

**Published:** 2020-01-01

**Authors:** Shen Shen, Dylan C. Dean, Zujiang Yu, Francis Hornicek, Quancheng Kan, Zhenfeng Duan

**Affiliations:** 1 Precision Medicine Center, The First Affiliated Hospital of Zhengzhou University, Zhengzhou, Henan 450052, China.; 2 Sarcoma Biology Laboratory, Department of Orthopaedic Surgery, David Geffen School of Medicine at University of Los Angeles, Los Angeles, CA 90095, USA.

**Keywords:** CDK9, chordoma tissues, LDC000067

## Abstract

**Objectives:** Chordomas are slow-growing malignancies that commonly affect vital neurological structures. These neoplasms are highly resistant to current chemotherapeutic regimens and often recur after surgical intervention. Therefore, there is an urgent need to identify molecular targets and more robust drugs to improve chordoma patient outcomes. It is well accepted that cyclin-dependent protein kinase 9 (CDK9) has tumorigenic roles in various cancers; however, the expression and significance of CDK9 in chordoma remains unknown.

Methods: Expression of CDK9 in chordoma cell lines and tumor tissues was examined by Western blot and immunohistochemistry (IHC). The correlation between CDK9 expression in patient tissues and clinical prognosis was analyzed. The functional roles of CDK9 in chordoma were investigated after the addition of small interfering RNA (siRNA) and CDK9 inhibitor (LDC000067). Cell growth and proliferation were assessed with MTT and clonogenic assays. The effect of CDK9 inhibition on chordoma cells was further evaluated with a three-dimensional (3D) cell culture model which mimics the* in vivo* environment.

**Results:** CDK9 was expressed in both chordoma cell lines and chordoma tissues. High- expression of CDK9 correlated with recurrence and poor outcomes for chordoma patients. CDK9 silencing with siRNA decreased growth and proliferation of chordoma cells and lowered levels of Mcl-1 and RNA polymerase II (RNAP II) phosphorylation. Pharmacological inhibition of CDK9 with the small molecular inhibitor LDC000067 reduced cell growth, supported apoptosis, suppressed cell colony formation in a clonogenic assay, and decreased spheroid growth in 3D culture.

**Conclusion:** We demonstrate that CDK9 expression in chordoma correlates with patient outcome, and, when inhibited, chordoma cell growth and proliferation significantly decreases. Taken together, these results support CDK9 as an emerging potential target in chordoma therapy.

## 1. Introduction

Chordomas are primary bone malignancies that arise along the axial skeleton and account for approximately 20% of primary spinal tumors and 3% of all bone tumors 1. Although chordomas are slow growing, they are aggressive and prone to metastasize. Their growth and pervasive nature can make complete surgical resection impractical, as they often compress critical neurological structures such as the brain, spinal cord, and nerves 2. Despite these surgical obstacles, resection remains the mainstay treatment as chordomas are highly chemoresistant. Currently, with thorough pre- and post-operative radiation therapy, the three and five year survival rates remain poor with high rates of relapse and metastasis 3. These limitations in current management highlight the urgent need to identify new molecular targets and chemotherapeutic strategies for chordoma.

Cyclin-dependent kinases (CDKs) are a family of serine/threonine protein kinases that regulate cell-cycle progression and DNA transcription 4. Previous studies have reported aberrant CDK expression and activation in various cancers 5-8. This has prompted a growing interest in novel CDK inhibitor drugs. One such drug, palbociclib (PD0332991), a CDK4/6 inhibitor, was approved by the Food and Drug Administration (FDA) for the treatment of breast cancer 9. With this clinical success, there have been an increasing number of clinical trials analyzing CDK inhibitors in various cancers 10-12.

Recently, CDK9 has seen more recognition for its role in DNA transcription initiation. Mechanistically, this occurs through a phosphorylation-mediated activation of the largest subunit of RNA polymerase II (RNAP II). This CDK9 mediated tumorigenic step has seen increasing attention within the literature and has been implicated in pancreatic cancer, hypopharyngeal carcinoma, leukemia, breast cancer, and hepatocellular carcinoma 13-17. However, the function and therapeutic potential of CDK9 in chordoma have not been elucidated. In this study, we show CDK9 is related to poor outcomes and promotes chordoma progression and inhibits apoptosis by regulating RNAP II and apoptosis-related proteins. These findings substantiate CDK9 as a potential novel target in chordoma treatment.

## 2. Materials and methods

### 2.1. Reagents

Human non-specific siRNA (5′-CUCUUAUCUACAUAAGGAU-3′) and CDK9 targeted siRNA (5'-GCUGCUAAUGUGCUUAUCA-3) were purchased from Sigma-Aldrich (St. Louis, MO). Lipofectamine® RNAiMAX was purchased from Thermo Fisher Scientific (Waltham, MA). The highly selective inhibitor of CDK9 (LDC000067) was purchased from Selleck Chemicals (Houston, TX). Monoclonal rabbit anti-CDK9, anti-p-RNAP II Ser-2 (13499S), anti-Survivin (2808S), anti-Bax (5023S), anti-Mcl-1(94296) and monoclonal mouse anti-α-tubulin (3874) antibodies were purchased from Cell Signaling Technology (Danvers, MA). The anti-RNAP II antibodies were purchased from Abcam (Cambridge, MA).

### 2.2. Chordoma tissue microarray (TMA)

A retrospective study with 55 tissue samples was conducted for the TMA immunohistochemical (IHC) stain as reported previously 18. These tissues were hematoxylin and eosin (H&E) stained to enable better evaluation and diagnosis of the chordoma tissue samples. All tissues were used in accordance with the policies of the institutional review board (IRB) of the hospital and common rules of the U.S. Department of Health and Human Services. Written informed consent was obtained from the patients for all specimens and clinical information used in this study. Histopathological and clinical information including patient age, gender, metastasis, recurrence, tumor location(s), months of follow-up, expression of brachyury, and disease status were collected (Table 1).

### 2.3. Immunohistochemistry (IHC)

The multi-tissue block was pre-heated for two hours at 60˚C. The sections were then deparaffinized in a series of xylene solutions, then rehydrated in graded ethanol and distilled water. Next, the slides underwent antigen retrieval with Target Retrieval Solution (Dako, North America, Inc., Carpinteria, CA) before being incubated in 3% hydrogen peroxide for 10 minutes to quench endogenous peroxidase activity. They were then blocked with 5% goat serum and incubated with human CDK9 primary antibody overnight in a 4°C humidified chamber (1:50 dilution, in 1% bovine serum albumin BSA. Cell Signaling Technology). After the slides were rinsed with Tris-buffered saline with Tween^®^ 20 (TBST) three times, the residual array bound antibodies were detected with SignalStain^®^ Boost Detection Reagent (Cell Signaling Technology) and SignalStain^®^ DAB (Cell Signaling Technology). The DAB reaction was terminated upon optimal color visualization. Following the DAB reaction, the slides were counterstained with hematoxylin and mounted with VectaMount AQ (Vector Laboratories, CA) for long-term preservation.

The stained slides underwent microscopic examination (Nikon Instruments Inc., NY) with the percentage of cells with positive nuclear staining calculated by two independent investigators blinded to the clinical data and the other viewer's score. The nuclear staining of CDK9 was graded into the following six groups: 0, <15% of cells stained positive; 1+, 15-30% stained positive; 2+, 31-50% stained positive; 3+, 51-70% stained positive; 4+, 71-85% stained positive; 5+, >85% stained positive.

### 2.4. Human chordoma cell lines and cell culture

The human chordoma cell line UCH1 and UCH2 were established and kindly provided by Dr. Silke Bruderlein (University Hospitals of Ulm, Ulm, Germany) 19. The cell line CH22 was established in our laboratory as previously reported 20. CH19, another new chordoma cell line, was established in our laboratory as well. The UCH2, CH22, and CH19 cells were maintained in Dulbecco's Modified Eagle Medium (DMEM) medium (GIBCO, Grand Island, NY) and the UCH1 cells were maintained in RPMI1640 medium (GIBCO). These media were supplemented with 10% fetal bovine serum (Sigma-Aldrich, MO) and 1% penicillin/streptomycin (Life Technologies, CA). In their respective media, the cells were incubated at 37°C with 5% CO_2_.

### 2.5. Immunofluorescence (IF) assay

The expression of CDK9 in chordoma cells was visualized via immunofluorescence assay as described previously 21. The UCH2 and CH22 cells were first seeded into 24-well plates before being incubated in 4% paraformaldehyde, fixed with ice-cold methanol, and blocked with 1% bovine serum albumin. Immunostaining was performed with CDK9 (1:200 dilution) and β-actin (1:500 dilution, Sigma-Aldrich, St. Louis, MO). After being washed three times with PBS, the cells were incubated with Alexa Fluor 594 (red) goat anti-mouse antibody and Alexa Fluor 488 (green) conjugated goat anti-rabbit antibody (Invitrogen) for one hour. Images were obtained with a Nikon Eclipse Ti-U fluorescence microscope equipped with a SPOT RT™ digital camera. Green color highlights CDK9 protein, and red color highlights the cytoplasm.

### 2.6. Western blotting analyses

Cell pellets and human chordoma tissues were lysed in protein lysis buffer. The protein concentrations were calculated with Protein Assay Reagents (Bio-Rad, CA) and a SpectraMax 340PC Microplate Reader from Molecular Devices (San Jose, CA). Equal amounts of protein were separated on 4-12% Bis-Tris gels (NuPAGE®, Thermo Fisher Scientific, CA) and transferred to nitrocellulose membranes, where they were incubated with specific primary antibodies at 4°C overnight (CDK9 1:1000 dilution; RNAP II at 1: 1000 dilution; p-RNAP II Ser-2 at 1: 1000 dilution; Mcl-1 at 1: 1000 dilution; Bax at 1: 1000 dilution; Survivin at 1: 1000 dilution α -Tubulin at 1: 1000 dilution). After being washed with TBST three separate times for five minutes, the membranes were further incubated with Goat anti-rabbit IRDye 800CW or Goat anti-mouse IRDye 680LT secondary antibody (LI-COR Biosciences, NE. Final images were obtained with Odyssey® CLx equipment (LI-COR Biosciences). The abundance of α-Tubulin was monitored to ensure equal loading.

### 2.7. MTT cell viability assay

Cell viability was determined by conventional 3-(4,5-dimethythiazol-2-yl)-2,5-diphenyl tetrazolium bromide (MTT) assay using a standard protocol as described previously. Treatment was performed with coincubation of UCH2 or CH22 cells with LDC000067 or siRNA for 120 hours. The color change was determined by a photometer set to a 490nm wavelength. The results were normalized and used to generate cell survival graphs with GraphPad Prism 7 software (GraphPad Software, CA).

### 2.8. Cell clonogenic assay

UCH2 and CH22 cell lines were seeded at 300 cells/well in 6-well plates with various concentrations of LDC000067 and 2mL medium. The medium was replaced every five days for fresh medium until there were visible colonies. These colonies were then Giemsa stained and manually counted. Pictures of the stained colonies were captured with a digital camera (Olympus, Tokyo, Japan).

### 2.9. 3D culture

CH22 and UCH2 cells were cultured in VitroGel^TM^ 3D (The Well Bioscience Inc., NJ) in 24-well plates at a density of 2×10^4^ cells/well, according to the manufacturer's protocol. Different cell culture media (with or without 5 µM of LDC000067) was added to cover the hydrogel. The plates were then placed in the incubator and had their cover media changed every 48 hours. Following a 21-day period, the spheroids were imaged on the Nikon Eclipse Ti-U fluorescence microscope equipped with a SPOT RT™ digital camera.

### 2.10. Statistical analysis

The data was analyzed using GraphPad PRISM 7 software. Statistical significance between groups was determined with a log-rank test for the Kaplan-Meier analysis, and a Wilcoxon signed-rank test for group comparisons. Errors were SD of averaged results and* p* values <0.05 were considered statistically significant between means.

## 3. Results

### 3.1. Correlations between CDK9 expression and clinical prognosis

**Figure 1:**
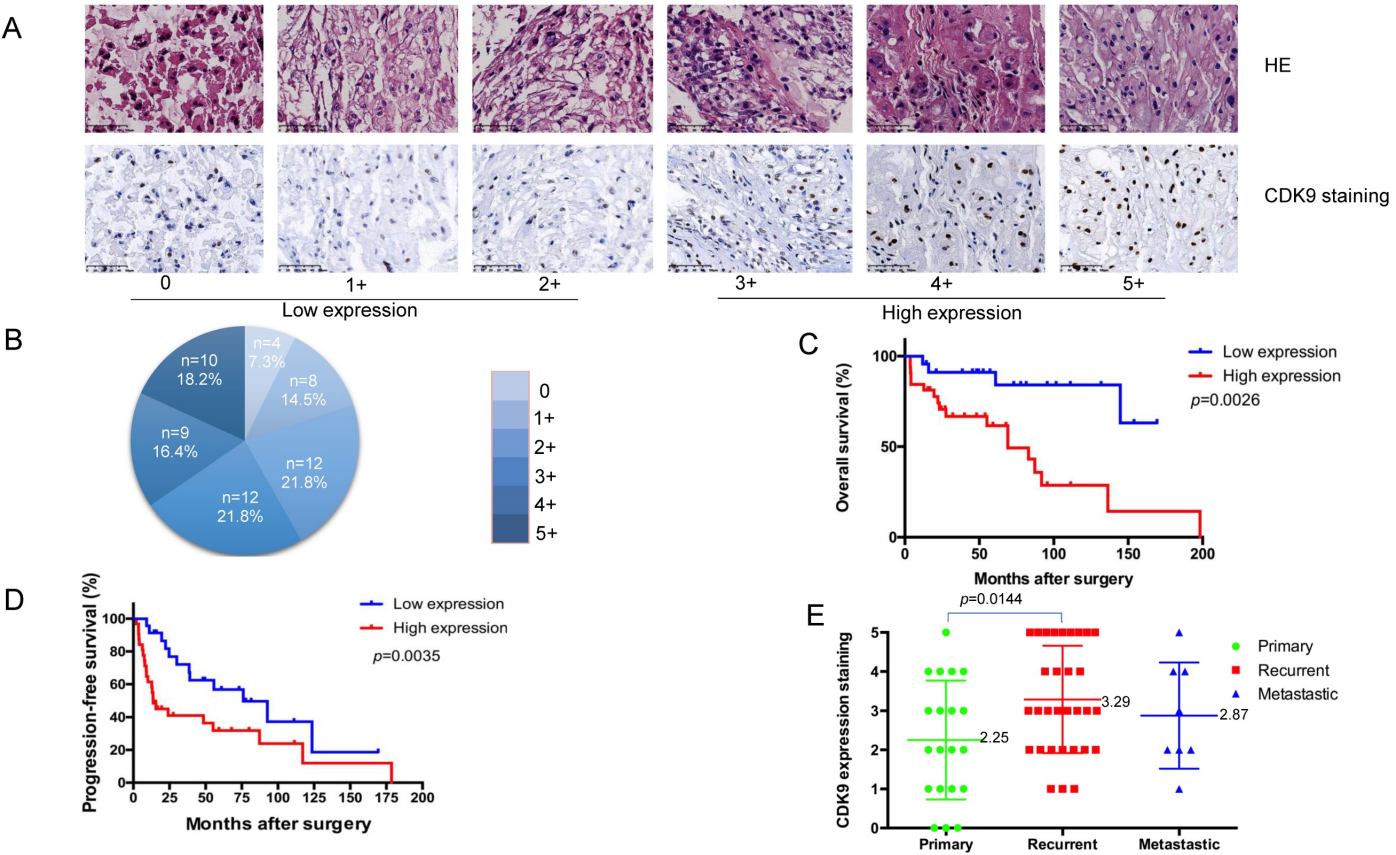
** Correlation between CDK9 expression and clinicopathological outcomes for chordoma patients. (A)** Representative images of different immunohistochemical stain intensities of CDK9. On the basis of the percentage of cells with positive nuclear staining, CDK9 staining patterns were categorized into 6 groups: 0, no nuclear staining; 1+: <10% of positive cells; 2+, 10%-25% of positive cells; 3+, 26%-50% of positive cells; 4+, 51%-75% of positive cells; 5+, >75% of positive cells. Original magnification 200×. **(B)** Pie chart representing relative frequency of different CDK9 staining patterns in chordoma tissue microarrays. (C) Kaplan-Meier curves depicting overall survival rates in the two groups of chordoma patients by CDK9 staining pattern. Low expression (number=24) mean OS = 78.4 months, high expression (number=31) mean OS = 50.2 months, p=0.0026. **(D)** Kaplan-Meier curves depicting progression-free survival rates in the two groups of chordoma patients by CDK9 staining pattern. Low expression (number=24) mean PFS= 57.5 months, high expression (number=31) mean PFS= 33.0 months, p=0.0035 (E) Levels of CDK9 expression in chordoma patients with primary chordoma (number=20, mean score=2.25), patients who developed recurrence (number=32, mean score=3.29) or metastasis (number=8, mean score=2.87).

To evaluate the clinical relevance of CDK9 expression in chordoma, we analyzed its expression in a human chordoma TMA. The CDK9 protein was predominantly localized within the nucleus of chordoma cells (Figure 1A). The patient characteristics of our cohort are summarized in Table 1. We analyzed 55 total patients who had an average age of 57.6 years (median age 59). Among these tissue samples, 20 (36.4%) were localized, 32 (58.2%) tissue samples were locally recurred only, and 3(5.4%) were metastatic without local recurrence. Of note, there were 8 total metastatic tissue samples, 5 of which had local recurrence and metastasis. The recurrent tissue samples were obtained from the primary chordoma site. IHC was conducted to assess the expression profile of CDK9. The samples were divided into two subgroups as follows: a low-expression group including 24 total patients with scores of 0 (4 of 55, 7.3%), 1+ (8 of 55, 14.5%), and 2+(12 of 55, 21.8%), and a high-expression group including 31 total patients with scores of 3+ (12 of 55, 21.8%), 4+ (9 of 55, 16.4%), and 5+ (10 of 55, 18.2%) (Figure 1A, 1B and Table 1).

Kaplan-Meier survival analysis revealed patients in the CDK9 low-expression group to have significantly better outcomes than those in the CDK9 high-expression group (p= 0.0026). Specifically, patients with a low-expression of CDK9 had a significantly longer overall survival (OS) time (mean OS = 78.4 months) compared to those with high-expression (mean OS = 50.2 months) (Figure 1C). Furthermore, CDK9 expression was inversely correlated with progression-free survival (PFS) (Figure 1D). The patients with low-expression of CDK9 had a longer PFS (mean PFS= 57.5 months) compared to the high-expression group (mean PFS= 33.0 months), which was statistically significant (*p*= 0.0035). The average CDK9 expression for patients with primary versus locally recurrent chordomas was statistically significant as well, at 2.25 and 3.29, respectively (*p*= 0.0144). The mean expression of CDK9 for the metastatic disease group was 2.87. There was no significant difference between the groups in terms of age, sex, location, or margin of resection (Table 2). There was also no significant difference between the metastatic disease group and the primary tumor or locally recurrent groups (Figure 1E). Taken together, these data outline how elevated CDK9 expression is a potential prognostic marker and agent of chordoma progression.

### 3.2. Expression of CDK9 in chordoma cell lines and tissues

**Figure 2:**
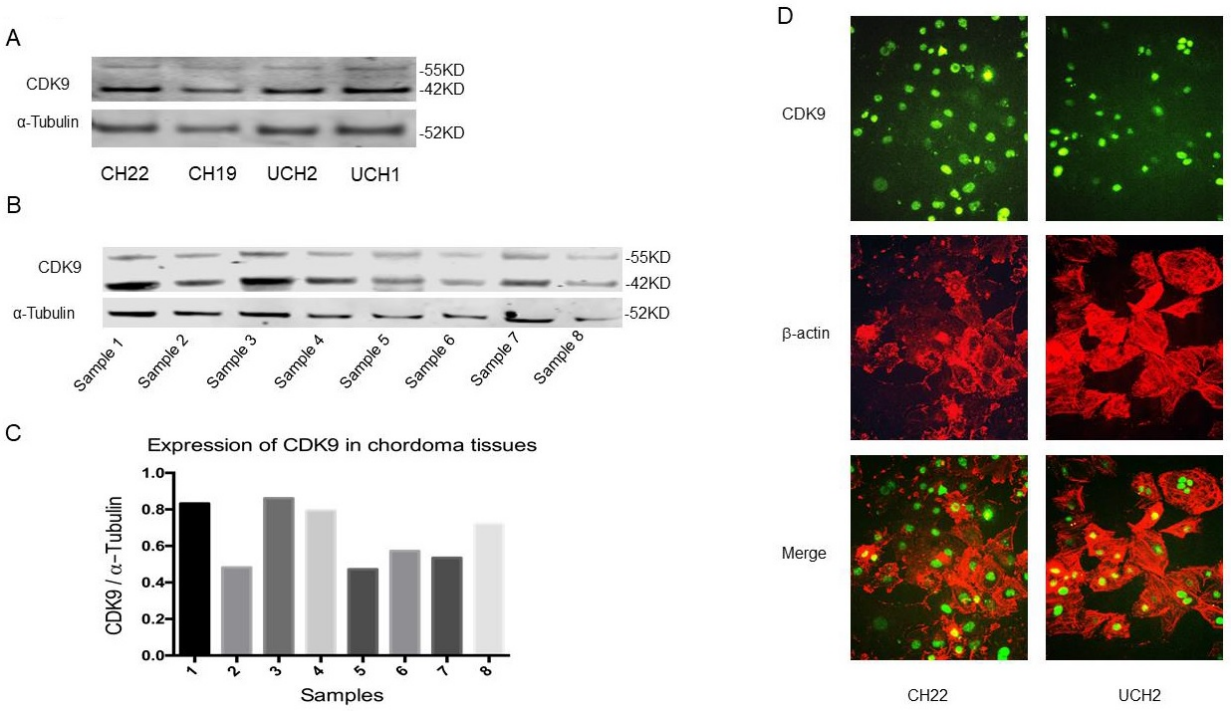
** CDK9 expression in chordoma cell lines and tissues. (A)** CDK9 expression in chordoma cell lines. **(B and C)** CDK9 expression in chordoma tissues. There are 2 isoforms of the CDK9 protein: the 42 KD CDK9 isoform and the 55 KD isoform. The smaller 42 KD isoform was the first identified isoform. Expression of CDK9 (55KD band) was normalized to α-Tubulin. **(D)** Cellular localization of CDK9 in UCH2 and CH22 cells was assessed by immunofluorescence with antibodies to CDK9 and actin.

CDK9 is expressed in two isoforms; a lighter 42KD isoform, and a heavier 55KD isoform which is transcribed from an upstream transcriptional start site which extends the shared mRNA sequence. This larger 55KD protein has an additional 117 amino acids at the N-terminus 22. Western blot analysis revealed both CDK9 isoforms are expressed in CH22, CH19, UCH2 and UCH1 cell lines (Figure 2A). To establish the existence of these two isoforms in primary cancer as well, we examined CDK9 expression in eight primary chordoma specimens. The tissues had variable levels of CDK9 expression (Figure 2B, 2C). Clinical information of the eight patients is shown in Supplementary Table S1.

To further confirm the expression of CDK9 and its subcellular localization, we tested CDK9 expression with immunofluorescence assay in CH22 and UCH2 cell lines. The CDK9 protein was localized in the nucleus in both chordoma cell lines (Figure 2D), which is consistent with the chordoma tissue IHC results (Figure 1A).

### 3.3. siRNA CDK9 inhibition in chordoma cells

**Figure 3:**
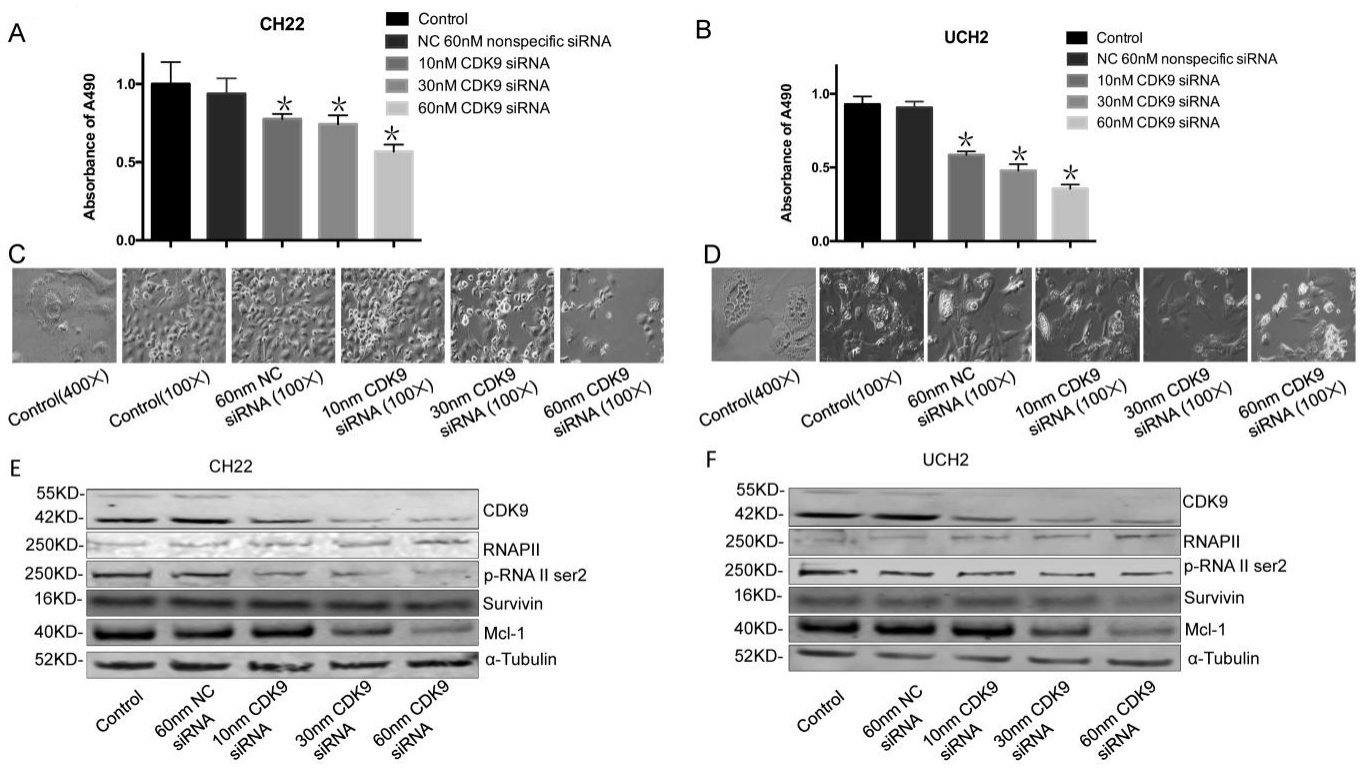
** Effect of CDK9 inhibition by siRNA on chordoma cell lines. (A and B)** After transfected with increasing concentrations of CDK9 specific siRNA or nonspecific siRNA for 3 days, cell viability was determined by MTT assay after siRNA transfection in both UCH2 and CH22 cells. *p<0.05. **(C and D)** The morphology of UCH2 and CH22 cell lines changed after 3 days of siRNA transfection. α-Tubulin was used as a loading control. **(E and F)** The proteins of CDK9 and downstream proteins p-RNA II ser2 and Mcl-1 in cells were examined by Western blot after 3 days of siRNA transfection.

To evaluate the functional role of CDK9 in chordoma, we used synthetic RNA interference (RNAi) to disrupt CDK9 expression in chordoma cells lines. The MTT assay showed CDK9 specific siRNA significantly inhibits cell growth and proliferation while non-specific siRNA has no significant effect (Figure 3A, 3B).

To evaluate how CDK9 silencing affects CDK9 regulated proteins, we analyzed the expression of RNAP II within CDK9 siRNA transfected chordoma cells. Additionally, we tested the efficacy of CDK9-siRNA in CH22 and UCH2 cell lines by Western blot. In brief, cells were incubated with 10 nM, 30 nM, and 60 nM CDK9 siRNA for 48 hours. As expected, CDK9-siRNA silencing occurred in a dose-dependent manner, while the untreated and nonspecific siRNA treated samples showed no significant effect (Figure 3E, 3F). Furthermore, there was a strong decrease of p-RNAP II Ser-2 expression in a dose-dependent manner (Figure 3E, 3F). Downregulation of Mcl-1 was also observed in the CDK9 siRNA transfected chordoma cells (Figure 3E, 3F). We found inhibition of CDK9 in chordoma cell lines to decrease p-RNAP II Ser-2 and Mcl-1 while also suppressing proliferation and inducing apoptosis. Collectively, these results suggest that inhibition of CDK9 suppresses growth and proliferation of chordoma cells.

### 3.4. Pharmacological CDK9 inhibition in chordoma cells

**Figure 4:**
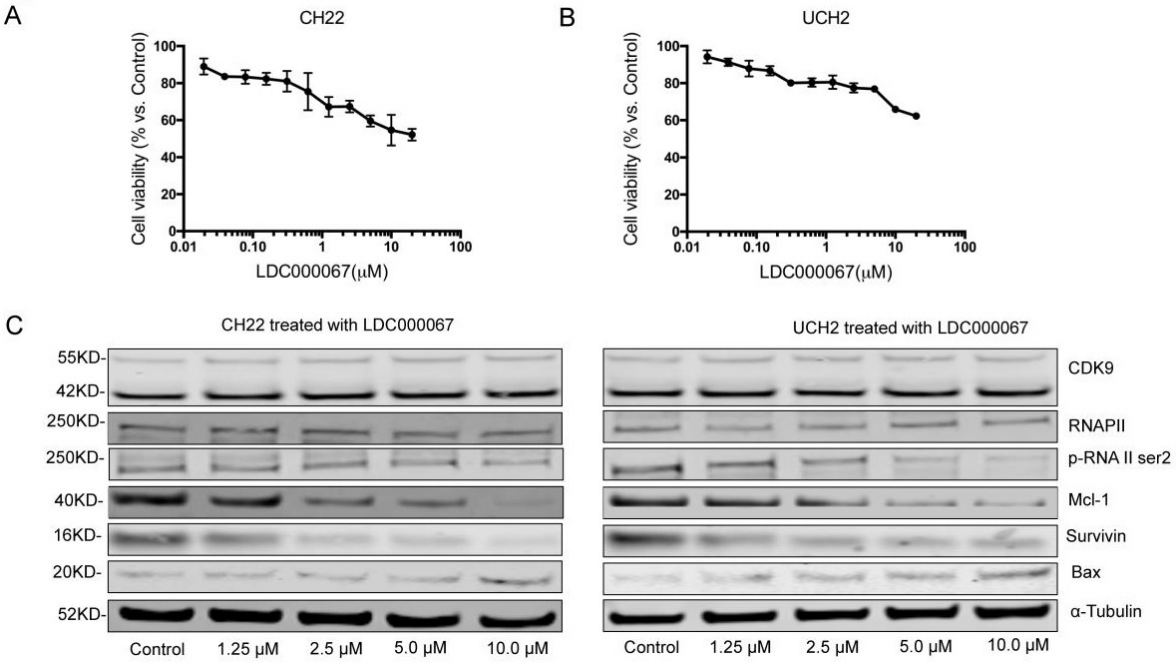
** Effect of CDK9 inhibitor treatment on chordoma cell lines. (A and B)** After exposure to increasing concentrations of CDK9 inhibitor for 120 h, cell viability was decreased in a dose-dependent manner in both UCH2 and CH22 cells, with the IC50 values for LDC000067 at 3.22 µM and 4.47 µM, respectively. **(C)** After incubation of UCH2 and CH22 cell lines with 1.25 µM, 2.5µM, 5.0µM, and 10.0 µM LDC000067 for 48 h, they showed a strong decrease of p-RNA II ser2 and Mcl-1 expression with increasing LDC000067 concentration. α-Tubulin was used as a loading control.

To investigate the effects of CDK9 inhibition at the protein level, we treated cells with the CDK9 inhibitor drug LDC000067. MTT assay confirmed a significant decrease in cell growth after LDC000067 treatment (Figure 4A, 4B). The half-maximal inhibitory concentrations (IC50) values for UCH2 and CH22 were 2.211μM and 1.354 μM, respectively.

To evaluate how CDK9 inhibition alters downstream pathways, we observed several CDK9 related downstream markers after LDC000067 treatment. Here we reveal that increasing concentrations of LDC000067 over a 48 hour period reduce p-RNAP II Ser-2 in a dose-dependent manner in both CH22 and UCH2 cells, without significantly affecting the CDK9 or RNAP II proteins. This finding is consistent with previous work which reports LDC000067 inhibits CDK9 activity without affecting its overall expression 23.

We also examined apoptosis-associated proteins in LDC000067 treated cells. As shown in Figure 4C, following LDC000067 treatment, expression of the pro-apoptotic protein Bax increased while the anti-apoptotic proteins Survivin and Mcl-1 decreased (Figure 4C). Overall, these results support targeting CDK9 as a potential strategy in chordoma therapy.

### 3.5. Effect of CDK9 inhibition on clonogenic assay and 3D culture

**Figure 5:**
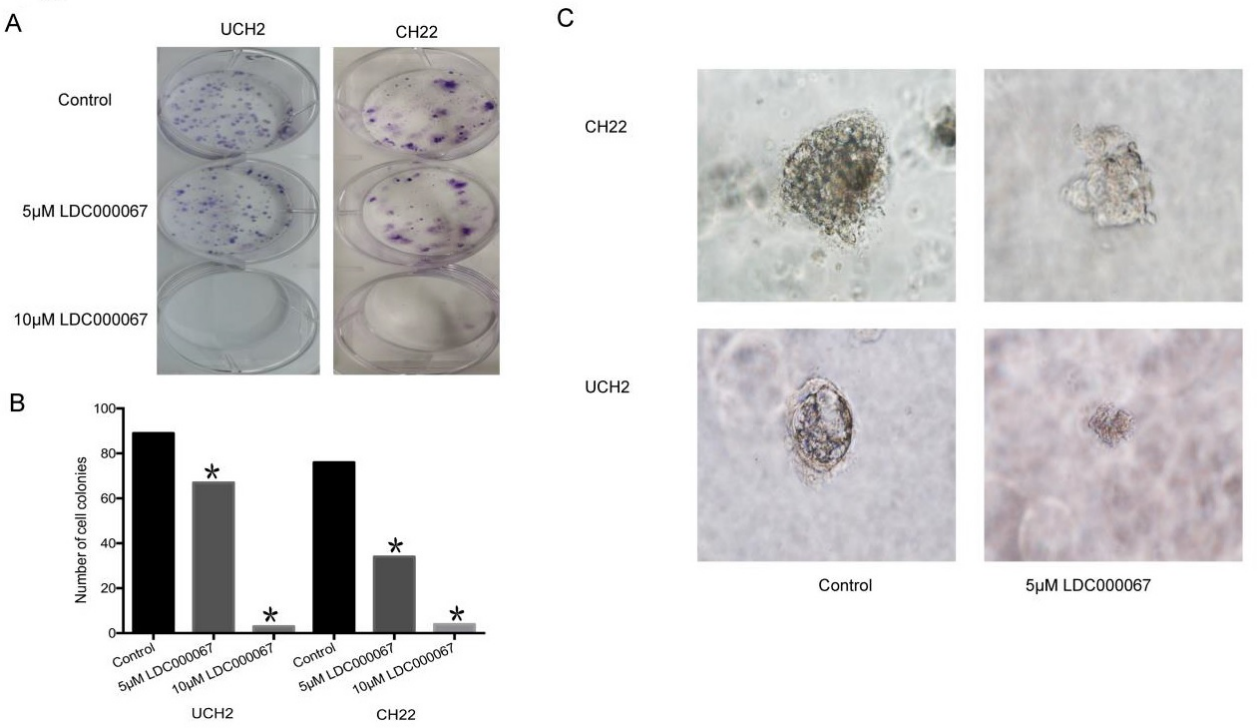
** Effects of CDK9 inhibition on clonogenic assay and 3D culture. (A and B)** Representative results of colony formation of chordoma cell lines treated with CDK9 inhibitor. **(C)** Assessed spheroid formation of chordoma cell lines treated with CDK9 inhibitor in 3D cell culture.

In order to assess the effect of CDK9 inhibition on colony formation, we implemented a clonogenic assay. The number and size of colonies in LDC000067 treated cells were significantly reduced, according to dose, compared to untreated cells (Figure 5A and 5B).

3D cell culture mimics the *in vivo* environment by permitting cancer cells to grow in all directions, similar to how they would in a living tissue. Given the advantage of this artificial environment, we investigated the effect of CDK9 inhibition on chordoma cell proliferation within the 3D culture. Over time, we observed the diameter of formed cancer spheroids from LDC000067 treated cells to be significantly smaller than the untreated cells (Figure 5C).

## 4. Discussion

Chordomas are resistant to radiotherapy and presently utilized drug regimens 24. Therefore, revealing which chemotherapeutics effectively target chordoma is crucial for advancing past the barriers of current chordoma therapies. In the present study, we show CDK9 is aberrantly expressed in chordoma cell lines and tumor tissues. In addition, the expression of CDK9 in patients with recurrent disease was significantly higher compared to those with primary disease. It seems likely, therefore, that CDK9 expression may feature more prominently in recurrent chordoma tumorigenesis. Further work is required to establish the exact mechanism of this relationship. This finding is especially important clinically, as recurrence remains a major obstacle in management and complete resection is complicated by the close relationship of these tumors with nearby neurovascular structures.

We observed that patients with a high expression of CDK9 have significantly worse outcomes in terms of OS and PFS. This significant correlation between CDK9 expression and patient outcome is consistent with previous works on other cancers and adds to a growing body of evidence recognizing CDK9 as a potential prognostic marker 13, 25, 26. For example, CDK9 can be highly expressed in pancreatic cancers, and when elevated, correlates to worse outcomes for these patients 13. Similar results have been reported in breast and gastric cancer 27. These findings, alongside our current work, provide increasing evidence that CDK9 may be an emerging biomarker with significant tumorigenic potential.

Recently, several studies have contended CDK9 targeting to be a promising chemotherapeutic strategy 13, 16, 17. Their promising results are likely a function of reducing the *activity* of CDK9 induced RNAP II transcription of various potentially tumorigenic genes. To verify the roles of CDK9 in chordoma cell growth and proliferation, we conducted a knockdown analysis using CDK9 specific siRNA. After this treatment, MTT revealed CDK9 knockdown to inhibit chordoma cell growth and proliferation. Furthermore, our Western blot data showed inhibition of CDK9 suppresses anti-apoptotic protein expression. Specifically, expression of Mcl-1 and Survivin decreased after genetic and chemical inhibition of CDK9.

CDK9 is known to transcriptionally upregulate the expression of Mcl-1 28, 29. In addition, previous works have shown inhibition of Mcl-1 to promote apoptosis and reduce tumor proliferation in Mcl-1 overexpressing osteosarcoma and melanoma cells 30, 31, prompting the preclinical development and clinical trials of Mcl-1 inhibitors 29, 32. Consistent with our work, we observed CDK9 inhibition to induce a concomitant decrease of Mcl-1 and associated antiapoptotic factors, with an overall suppression of chordoma cell growth. Survivin is a member of the inhibitor of apoptosis protein family (IAP) and functionally suppresses the terminal effector enzymes caspase-3 and caspase-7 33, 34. Experimental findings have revealed inhibition of Survivin to prevent chordoma cell growth, and as expected, to induce apoptosis 35. Collectively, the decrease in Mcl-1 and Survivin reasonably explains how inhibition of CDK9 may induce apoptosis in chordoma cell lines.

To expand the clinical application of our work and further characterize the role of CDK9 in chordoma cell growth and proliferation, we conducted an inhibition analysis using the specific CDK9 inhibitor LDC000067. This novel drug specifically binds to the ATP binding pockets of kinases. Importantly, the selectivity of LDC000067 for CDK9 is better than other CDK9 inhibitors such as flavopiridol and DRB, making it ideal for our investigation 23. In our study, the addition of LDC000067 in chordoma cell lines showed encouraging results, as it inhibited tumor cell growth and proliferation. In accordance with our work, previous studies have demonstrated that pharmacological inhibition of CDK9 induces apoptosis in several human cancers 13, 23, 36. For example, at concentrations of LDC000067 greater than 2 μM, breast cancer cell lines increase their percentage of apoptotic cells 23. Our study shows the expression of Survivin to decrease and the expression of the pro-apoptotic protein Bax to increase in LDC000067 treated chordoma cells, suggesting this drug can disrupt aberrant CDK9 apoptotic signaling cascades. As an additional mechanism, phosphorylation of the RNAP II CTD residues serine 2 (p-RNA II ser2) was inhibited in a dose-dependent manner after treatment with CDK9 inhibitor, which is consistent with previous reports on colon carcinoma and adult T-cell leukemia/lymphoma 15, 37. Overall, our results support LDC000067 mediated inhibition of CDK9 as a potential strategy in chordoma therapy.

Finally, we utilized a 3D cell culture platform to better connect our *in vitro* work with *in vivo* application. Permitting cancer to form spheroids allows for more accurate assessment of chemotherapeutic agents, as the cancer model better mimics live tissue drug diffusion, retention, and therapeutic outcome 38. We successfully established 3D spheroids of chordoma cells and found that CDK9 inhibition significantly diminishes chordoma spheroid growth.

In summary, our study demonstrates that CDK9 is highly expressed in chordoma, and when elevated, correlates with recurrence and worse prognosis. We also show knockdown and inhibition of CDK9 decreases chordoma cell proliferation and growth. Although there is abundant room for further research and validation, these findings suggest CDK9 is a potential therapeutic target for chordoma treatment.

## Supplementary Material

Supplementary figures and tables.

## Figures and Tables

**Table 1:** Correlations between CDK9 expression and clinicopathological features

Parameter		Number of cases (%)	CDK9 low expression (n, %)	CDK9 high expression (n, %)
Total		55	24(43.6%)	31(56.4%)
Age	Average	57.6	61.22	55.00
	Median age	59	62	52
Sex	Male	41	18(43.9%)	23(56.1%)
	Female	14	6(42.9%)	8(57.1%)
Location	Mobile Spine	21	8(38.1%)	13 (61.9%)
	Sacral	34	16(47.1%)	18(52.9%)
Stage	Primary	20	11(55.0%)	9 (45.0%)
	Metastatic	8	4(50.0%)	4(50.0%)
	Recurrent	32	11(34.4%)	21(65.6%)
Status	NED	22	10(45.5%)	12(54.5%)
	AWD	8	7(87.5%)	1(12.5%)
	DOD	23	5(21.7%)	18(78.3%)
	DOO	2	2(100.0%)	0(0.00%)

NED: No evidence of disease, AWD: Alive with disease, DOD: Dead of disease, DOO: Dead of other disease.

**Table 2:** The clinical parameters of chordoma tissue microarray

Variables	Total number of patient events	Non-survival n (%)	Survival (month) Mantel-Cox analysis
Age at diagnosis, year			0.4296
<50	19	8(42.1%)	
50	36	14(38.9%)	
Sex			0.5404
Male	41	16(39.0%)	
Female	14	6(42.8%)	
Location			0.7308
Mobile Spine	21	11(52.4%)	
Sarcral	34	12(35.3%)	
Margin			0.0101
Intralesional	23	15(65.2%)	
Marginal	3	2(66.7%)	
Attempted wide with contanminated margin	10	1(10.0%)	
Wide	16	3(18.8%)	
N/A	3	1(33.3%)	
Radiation			0.4430
No	12	6(50.0%)	
Yes	43	16(37.2%)	
Brachyury			0.5367
Low	30	14(46.7%)	
High	18	6(33.3%)	
Unknown	7	2(28.6%)	
CDK9 expression			0.0026*
Low	24	4(16.7%)	
High	31	18(58.1%)	

N/A: not applicable. *Statistically significant
