# Supplementary material for: Aberrant CDK9 expression within chordoma tissues and the therapeutic potential of a selective CDK9 inhibitor LDC000067
Source: J Cancer. 2020 Jan 1;11(1):132–41. doi: 10.7150/jca.35426 (PMC6930393; doi:10.7150/jca.35426)
Supplement: Supplementary file 1 — Supplementary figures and tables. [file jcav11p0132s1.pdf]

Supplementary figure- Figure S1

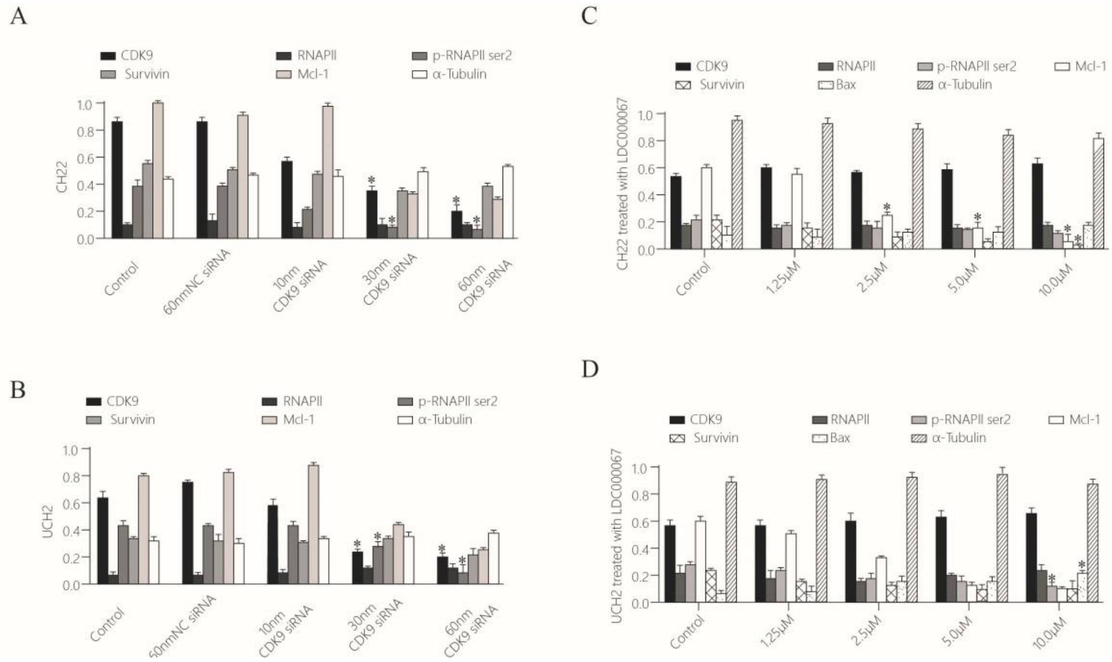

FIGURE S1. The adequate contrast and clarity of the gel bands

(A and B) The proteins of CDK9 and downstream proteins p-RNA II ser2 and Mcl-1 in cells were examined by Western blot after 3 days of siRNA transfection. (C and D) After incubation of UCH2 and CH22 cell lines with 1.25 µM, 2.5µM, 5.0µM, and 10.0 µM LDC000067 for 48 h, they showed a strong decrease of p-RNA II ser2 and Mcl-1 expression with increasing LDC000067 concentration. α-Tubulin was used as a loading control.

Supplementary Table S1. Clinical data of protein samples from chordoma tissues

Sample	location	Follow-up (month)	Metastasis	Recurrence	Status of final follow-up
1	sacrum	N/A	no	no	Non-survival
2	N/A	N/A	no	no	Survival
3	sacrum	N/A	no	no	Survival
4	sacrum	87.8	no	no	Non-survival
5	sacrum	28	no	yes	Non-survival
6	sacrum	N/A	no	yes	Survival
7	sacrum	N/A	yes	no	Survival
8	sacrum	118	no	yes	Survival
